# The influence of pediatric cancer treatment on taste perception and food hedonics: a systematic review

**DOI:** 10.1093/nutrit/nuad162

**Published:** 2024-01-10

**Authors:** Annie R Curtis, Sze Yen Tan, Anna Boltong, Jennifer Cohen, Nicole Kiss

**Affiliations:** Institute for Physical Activity and Nutrition, Deakin University, Geelong, Victoria, Australia; Institute for Physical Activity and Nutrition, Deakin University, Geelong, Victoria, Australia; Cancer Australia, Surry Hills, New South Wales, Australia; Kirby Institute, University of New South Wales (UNSW) Medicine, UNSW, Kensington, New South Wales, Australia; Discipline of Paediatrics and Child Health, UNSW Medicine & Health, Randwick Clinical Campus, UNSW Sydney, Sydney, New South Wales, Australia; Canteen Australia, Sydney, New South Wales, Australia; Institute for Physical Activity and Nutrition, Deakin University, Geelong, Victoria, Australia; Allied Health Department, Peter MacCallum Cancer Centre, Melbourne, Victoria, Australia

**Keywords:** chemotherapy, hedonics, pediatric cancer, radiotherapy, taste

## Abstract

**Context:**

Children with cancer are at risk of poor nutritional status during treatment and into survivorship. Objectively measured taste perception and self-reported food hedonics are 2 factors that may influence food intake.

**Objective:**

This 2-armed systematic review examined whether chemotherapy and radiotherapy affect (1) taste perception and (2) hedonic experiences of children and survivors of childhood cancer.

**Data Source:**

A 2-armed systematic literature search was conducted in the Medline, CINAHL, Embase, and PsychInfo database until June 2022. The effects of cancer treatment on objective taste perception or food hedonics (ie, food liking or aversion and appetite) were examined.

**Data Extraction:**

Peer-reviewed articles published in English of studies that included children (aged <18 years) or survivors of childhood cancer (any age) were reviewed. Risk of bias was determined using the Evidence Analysis Library by the Academy of Nutrition and Dietetics.

**Data Analysis:**

A total of 1417 articles in the taste search arm and 3862 articles in the hedonics search arm were identified. Of these, 9 and 4 articles were eligible for review, respectively. Cancer treatment had highly variable effects on taste perception during treatment and into survivorship. Learned food aversions were experienced by children receiving chemotherapy treatment and liking of meats and salty foods by children with cancer was affected. The impact of treatment on appetite varied.

**Conclusions:**

Cancer treatment did not uniformly affect taste perception. Food liking may be negatively affected, and learned food aversions may develop during cancer treatment. To establish the clinical relevance of childhood cancer treatment on taste perception and food hedonics, more research is required.

**Systematic Review Registration:**

PROSPERO registration no.CRD42020207127.

## INTRODUCTION

Malnutrition affects up to 75% of children and adolescents with cancer.^1^ Children who are malnourished experience a 2-fold increased risk of death.^2^ Optimal nutritional status during and after cancer treatment is associated with improved survival, better quality of life, and a reduction in the risk of infection and chronic diseases.^3–7^ Adequate dietary intake is integral to maintaining nutritional status. However, dietary intake is adversely affected by treatment-related side effects, including changes in taste perception and the hedonic experience of food (ie, the enjoyment of food), that contribute to the etiology of malnutrition.^8^

Taste is the sensation perceived when chemical molecules stimulate receptor cells within the oral cavity.^9^ Taste receptor cells rapidly divide, making them susceptible to the effects of cancer treatment.^10–12^ It is estimated that up to 60% of children experience altered taste perception during cancer treatment,^13^ with effects remaining into survivorship.^14^ In children and adults undergoing chemotherapy, altered taste perception has been linked to difficulties in maintaining dietary intake,^8^^,^^15^ including decreased energy and nutrient intakes,^16^ which may result in the development of malnutrition. The terms “taste” and “flavor” are often used interchangeably in practice; however, this is a conflation of terms.^17^ Flavor is a multisensory experience encompassing taste, smell, touch, chemesthesis, food appearance, and audition.^18^^,^^19^

Taste is 1 of many factors that defines our eating experience, or food hedonics. The sensory (ie, taste) and hedonic systems are separate but related entities. By definition, hedonics refers to the psychological determination of the extent to which a life experience is pleasant or unpleasant.^20^ In the context of dietary intake, hedonics define the extent to which eating evokes enjoyment and “food liking” or displeasure and “food aversions.”^21^ These reactions are associated with physiological and psychological functions, including appetite, hunger, and learned food aversions.^22^ These functions of hedonic experience can be negatively affected by cancer treatment; for example, reduced appetite is commonly experienced by both adults and children throughout cancer treatments,^17^^,^^23^ limiting the patients drive to eat. Learned food aversions are the perceived associations between specific foods or taste qualities and unpleasant symptoms.^24^ Learned food aversions were first linked to chemotherapy through negative associations or nausea experienced as a side effect of chemotherapy.^25^

Previous systematic reviews have examined cancer treatments and associations with altered taste perception and hedonic factors such as food liking and appetite in adults.^17^^,^^26^^,^^27^ Chemotherapy was shown to have variable influences on taste function and an adverse effect on food liking and appetite in adults with cancer.^17^ However, associations between cancer treatment and taste or food hedonics have yet to be thoroughly investigated in children. A comprehensive understanding of how cancer treatment may affect taste perception and food hedonics, such as food liking, food aversion, or appetite, is essential to develop effective assessment and diagnostic frameworks and timely nutritional interventions for the prevention and treatment of malnutrition and achievement of nutritional requirements for childhood growth and development. As such, in this 2-armed systematic review, we aimed to determine the effects of childhood cancer treatment on (1) taste perception and (2) hedonic factors, including food liking, food aversion, and appetite, among children with cancer and childhood cancer survivors.

## METHODS

Because of the interconnected nature of taste perception and hedonic factors such as food liking and appetite, we examined these concepts in parallel in this review. This 2-armed systematic review was conducted in accordance with reporting requirements of the Preferred Reporting Items for Systematic Reviews and Meta-analysis (PRISMA) updated guideline for reporting systematic reviews (see Table S1 in the Supporting Information online).^28^ A research protocol was registered with the International Prospective Register of Systematic Reviews in October 2020 (registration no. CRD42020207127). The chosen search strategy was adapted from a 2012 review by Boltong and Keast^17^ that examined the influence of chemotherapy on taste perception and hedonic experience in adults with cancer.

### Data sources and searches

Two systematic literature searches (the taste and hedonics search arms) were conducted in the Medline Complete, Embase, CINAHL Complete, and PsychInfo databases from database inception to June 21, 2022. Two independent searches were conducted to fully capture each concept (taste and hedonics) separately, because these concepts are not exclusively examined together in the literature. The search strategy was developed in consultation with all authors and an experienced health sciences librarian to improve scientific rigor. Literature was searched by title and abstract. For the taste search arm, key search terms were “chemotherapy,” “radiotherapy,” and “taste.” For the hedonics search arm, search terms were “chemotherapy,” “radiotherapy,” “liking,” “appetite,” and “hedonic*.” Medical Subject Headings were applied for each search term. Limits included “humans,” “peer-review,” and “English language.” The full search as conducted in Medline Complete is outlined in Tables S2 and S3 in the Supporting Information online. A snowball search was conducted by 1 author (A.R.C.). Reference lists of included articles were examined to identify relevant articles not uncovered by the literature search. Key studies already known to the authors were also included.

### Study selection

Articles published in peer-reviewed journals were eligible for this review if they were published in English and reported on studies involving children aged ≤18 years who were undergoing chemotherapy and/or radiotherapy, including conditioning therapy as part of blood or marrow transplant (BMT) protocols, for cancer at the time of analysis. To assess the burden of treatment on survivors of childhood cancer, children or adults who received treatment as a child, with no additional treatment as an adult, were also included.

For the taste search arm, eligible articles investigated taste perception during and/or after cancer treatment. Taste was defined as the perception of the 5 known taste qualities (sweet, sour, salty, bitter, and umami) and was assessed as a function of taste sensitivity (taste detection and/or recognition thresholds) or perceived taste intensity, using validated objective measurement tools. Articles reporting on work in which taste was examined using subjective methods, including self-reported experience or parent observations, were excluded due to the variability in reporting methods. For the hedonics search arm, eligible articles examined the broad components of hedonics (food liking, aversions, and appetite) as a direct effect of cancer treatment. Only articles that included a comparison to an internal reference (ie, change in hedonic experience since commencing treatment) or control group such as healthy children or normative data were eligible. Table 1 presents the PICOS criteria for study selection.

**Table 1:** PICOS criteria for inclusion of studies

Criterion	Details
Participants	Children (aged <18 y) undergoing cancer treatment at the time of analysis or survivors of childhood cancer who received cancer treatment as a child
Interventions	Chemotherapy or radiotherapy
Comparisons	Children without cancer or before and after cancer treatment
Outcomes	Objectively assessed taste function and food hedonics
Study design	Any study design

Identified articles were assessed against the outlined eligibility criteria in 2 stages. Initially, study titles and abstracts were screened independently by three reviewers (A.R.C. and two assistants). Articles that unanimously met the eligibility criteria or provided insufficient information within the title or abstract to be assessed with certainty were progressed to the next screening phase. Relevant full-text articles were independently screened against the eligibility criteria by three reviewers (A.R.C. and two assistants). Conflicts were resolved via verbal discussion between reviewers; when agreement could not be reached, a fourth author (N.K.) was consulted.

### Data extraction and quality assessment

Data extraction charts were developed by 1 author (A.R.C.) on the basis of those produced by Boltong et al^17^ for a systematic review of the same kind in adults. Extracted information included study design, participant, and comparator details; taste or hedonics parameters; assessment tools; and study findings. Data extraction was conducted in Microsoft Excel by 1 author (A.R.C.) and verified by a second author (T.P. or N.K.). Quality appraisal was independently performed by 3 authors (A.R.C., N.K., S.Y.T.) using the Academy of Nutrition and Dietetics Evidence Analysis Library Quality Criteria Checklist: Primary Research.^29^ All included articles were assessed based on relevancy and validity and were ultimately rated positive, neutral, or negative. Eligible articles were included in this review regardless of the quality criteria assigned.

## RESULTS

### Literature search results

**Figure 1:**
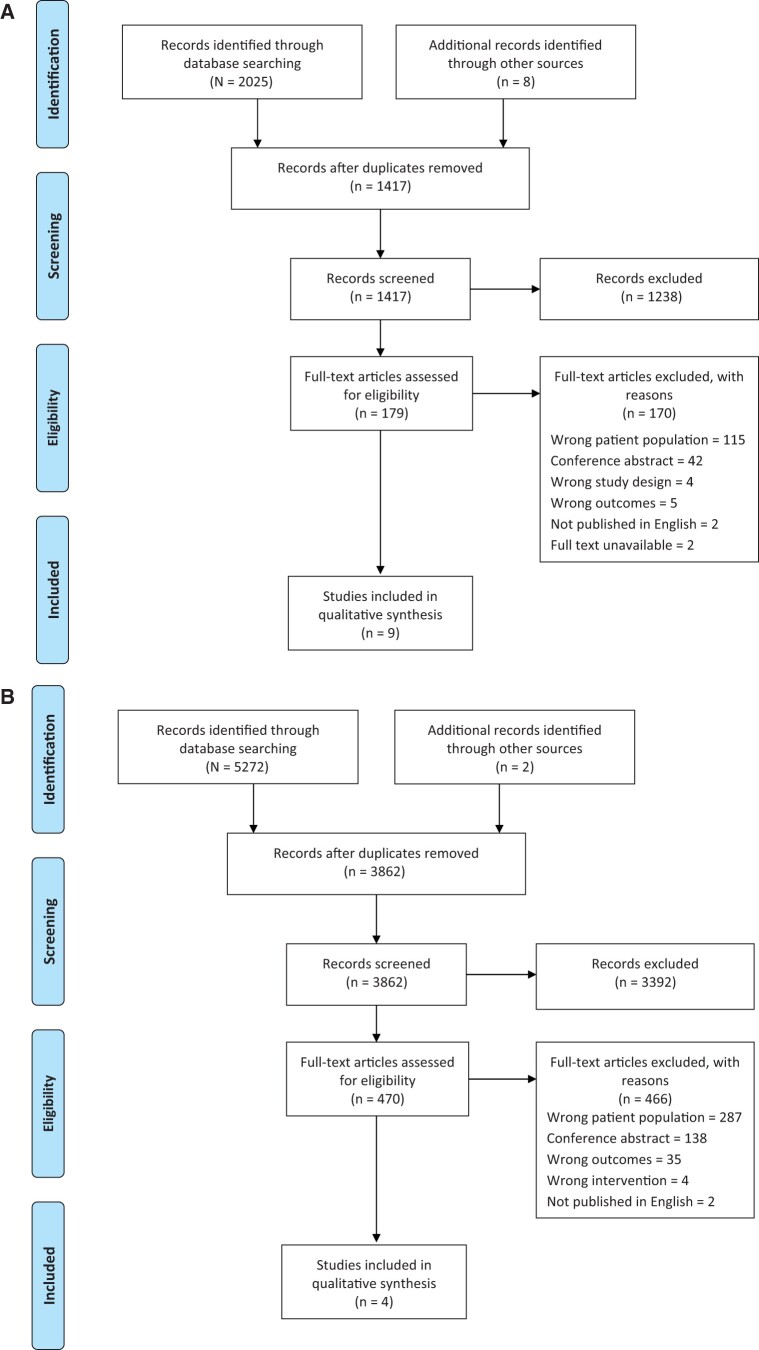
Flow diagram depicting the main stages of the systematic review: taste arm (A) and hedonics arm (B).

In total, 1417 and 3862 studies were screened in the taste (Figure 1A) and hedonics (Figure 1B) arms of this review, respectively. Nine taste studies and 4 hedonics studies met the inclusion criteria and were included. One article was included in both arms of this review.

### Quality assessment

Outcomes of the quality assessments are reported in Table 2.^8^^,^^14^^,^^23^^,^^25^^,^^30–37^ Of the 12 included studies, 6 were rated positive, 3 were rated neutral, and 3 rated negative.

**Table 2:** **Quality assessment of included studies (n = 12) using the Academy of Nutrition and Dietetics Evidence Analysis Library Quality Criteria Checklist: Primary Research** ^29^

Reference		Validity questions									
	Quality rating	1. Research question	2. Subject selection	3. Study groups	4. Withdrawal handling	5. Blinding	6. Interventions described	7. Outcomes defined	8. Statistical analysis	9. Conclusions	10. Funding bias
Ameringer et al, 2013^23^	+	Yes	Yes	Yes	Yes	Yes	Yes	Yes	Unclear	Yes	Yes
Barale et al, 1982^30^	∅	Yes	No	Unclear	Unclear	Yes	Yes	Yes	Unclear	Unclear	Yes
Bernstein et al, 1978^25^	–	Yes	No	Unclear	Unclear	No	Unclear	Unclear	No	Unclear	Unclear
Bernstein et al, 1982^31^	–	Unclear	No	Unclear	Unclear	No	No	Yes	Unclear	No	Unclear
Cohen et al, 2012^32^	+	Yes	Yes	N/A	Unclear	Yes	Yes	Yes	Unclear	Yes	Yes
Cohen et al, 2014^14^	∅	Yes	Yes	No	N/A	Yes	No	Unclear	Yes	Yes	Yes
Majorana et al, 2015^33^	∅	Yes	Unclear	N/A	Yes	Yes	Yes	Yes	Unclear	Yes	Yes
Nagai et al, 2014^34^	+	Yes	Yes	Yes	Yes	Yes	Yes	Yes	Unclear	Yes	Yes
Skolin et al, 2006^8^	+	Yes	Yes	Yes	N/A	Yes	Yes	Yes	Yes	Yes	Yes
Tanem et al, 2022^35^	+	Yes	Yes	No	Yes	Yes	Yes	Yes	Unclear	Yes	Yes
Trogdon Wall and Gabriel, 1983^36^	–	Yes	Unclear	Unclear	Yes	Unclear	No	Unclear	Unclear	Unclear	Yes
Van den Brink et al, 2021^37^	+	Yes	Yes	Yes	Yes	Yes	Yes	Yes	Unclear	Yes	Yes

*Abbreviation*: N/A, not applicable. *Symbols*: +, positive; –, negative; ∅, neutral.

### Study characteristics

In the taste search arm, data from 139 children with cancer and 163 survivors of childhood cancer were reported by nine studies (Table 3^8^^,^^14^^,^^30^^,^^32–37^). Approximately 70% of all cancer types were hematological. Chemotherapy^8^^,^^36^^,^^37^ or BMT^30^^,^^32^^,^^33^ was the primary treatment modality in each of 3 articles, respectively. Three articles reported on studies that included patients receiving chemotherapy, radiotherapy, hematopoietic stem cell transplant, or a combination of these.^14^^,^^34^^,^^35^ Taste perception was assessed using detection or recognition thresholds in all of the included studies. Majorana et al^33^ also assessed taste intensity. Taste perception was assessed during cancer treatment in 6 studies,^8^^,^^30^^,^^32^^,^^33^^,^^36^^,^^37^ and 3 articles described researchers’ examination of taste perception in survivors of childhood cancer between 6 months and 40 years after treatment.^14^^,^^34^^,^^35^ Most studies compared taste perceptions between children with cancer or survivors, with control groups without cancer or normative data. In 4 articles, authors further examined changes in taste perception across a treatment cycle (chemotherapy or transplant).^30^^,^^32^^,^^33^^,^^37^

**Table 3:** Characteristics and findings from studies that investigated taste perception in children with cancer and in cancer survivors

Reference; study design	Country	Participants and cancer type	Treatment	Control	Objective taste measurement and tastant	Key findings
Barale et al, 1982^30^ Prospective cohort design	United States	Children receiving treatment (n = 11); aged 6–15 y ALL, AML, AMML Number of children with each cancer type was not specified.	Marrow transplantation: intrathecal methotrexate and cyclophosphamide, 60 mg/kg, followed by 1000 to 1575 rad total body irradiation. Methotrexate given posttransplant for the first 100 d.	Sex-matched and age-similar siblings, unrelated participants from families on the hospital unit, and community (n = 20) Comparisons were also made among admission, day 2, and day 45 after transplant (own control).	Recognition threshold of sweet, sour, salt, bitter	Patients had lower sensitivity to sour than did control participants on admission. No significant between-group differences were observed for sweet, salt, or bitter tastes.
Cohen et al, 2012^32^ Prospective cross-sectional cohort design	Australia	Children receiving treatment (n = 10); mean age: 11.2 y (allogenic transplant) and 13.8 y (autologous transplant) High-risk ALL (n = 3); AML (n = 1); relapsed ALL (n = 3); Hodgkin’s lymphoma (n = 2); biphenotypic leukemia (n = 1)	Allogenic and autologous transplant Types of allogenic transplant: matched sibling (n = 3); matched, unrelated donor (peripheral blood stem cell; n = 2); cord blood (n = 2) Conditioning regimes: Allogenic transplant: thiotepa, cyclophosphamide, ATGAM (Pfizer) (n = 7); total body irradiation (n = 7) Autologous transplant: etoposide, cyclophosphamide (n = 1); cyclophosphamide, carmustine/etoposide (n = 1); melphalan (n = 1)	Comparisons were made at admission and monthly thereafter. Gustatory dysfunction was determined if ≥ 3 concentrations of a tastant were incorrectly identified based on normative child data.	Detection and recognition threshold for sweet (sucrose), sour (citric acid), salt (sodium chloride), bitter (quinine hydrochloride)	No significant differences for total taste scores or sweet, sour, salt, or bitter, between baseline and follow up. At 1 mo after transplant, 3 participants experienced taste dysfunction (sour, n = 2; bitter, n = 1). No evidence of taste dysfunction at 2 mo after transplant
Cohen et al, 2014^14^ Cross-sectional design	Australia	Childhood cancer survivors^a^ (n = 50); mean age: 19.7 y ALL (n = 18); AML (n = 1); neuroblastoma (n = 4); Wilms’ tumor (n = 4); rhabdomyosarcoma (n = 3); lymphoma (n = 4); medulloblastoma (n = 2); Ewing’s sarcoma (n = 2); osteosarcoma (n = 3); other (n = 10)	Chemotherapy (n = 27); chemoradiotherapy (n = 17) Radiotherapy: cranial (n = 6); abdominal (n = 2); head and neck (n = 1); other sites (n = 8); total body irradiation (n = 4) HSCT (n = 7) Chemotherapy type not specified	Identification of < 4 of 5 tastant concentrations was considered an impaired ability to detect that taste, based on normative data; children older than 5 y (n = 232) and adults (n = 56)	Recognition threshold for sweet (sucrose), sour (citric acid), salt (sodium chloride), bitter (quinine hydrochloride)	Taste dysfunction was experienced by 27.5% of participants (9.8% for sweet; 15.7% for sour; 7.8% for salt; 11.8% for bitter). No significant differences were found for total scores for sweet, sour, salty, bitter, and water (*P* = 0.490). No significant relationships between taste scores and the age at diagnosis (*P* = 0.585) or years since treatment completion (*P* = 0.481) were found.
Majorana et al, 2015^33^ Prospective design	Italy	Children receiving treatment (n = 51); mean age: 5.2 y Cancer type: hemato-oncologic diseases, further details not specified	HSCT Conditioning regimes: antithymocyte globulin, busulfan, cyclophosphamide, alemtuzumab, fludarabine, melphalan, thiotepa, vepeside	Comparisons were made between pretreatment, during conditioning therapy, and every 3 mo after engraftment (own control).	Detection threshold and taste intensity: sweet (sucrose); sour (citric acid); salt (sodium chloride); bitter (quinine hydrochloride)	All tastants were detected at the lowest concentration at pretreatment. Bitter, sour, and salt tastes were detected at the second highest concentration and sweet at the third highest concentration during conditioning therapy. After engraftment, bitter and sweet tastes were detected at the lowest concentration. Sour and salt tastes remained undetected until the third highest concentration. Intensity ratings did not differ between pretreatment and after engraftment (*P* = 014).
Nagai et al, 2014^34^ Cross-sectional design	Japan	Childhood cancer survivors^b^ (n = 73); mean age: 13 y (range, 7–18) ALL (n = 46); AML (n = 4); lymphoma (n = 8); Langerhans cell histiocytosis (n = 3); brain tumor (n = 5) (6.8); others (n = 7)	Chemotherapy (n = 40); chemotherapy + radiation (n = 8); chemotherapy + HSCT (n = 5); chemotherapy + radiation: + HSCT (n = 20); and chemotherapy type not specified	Healthy elementary school children (n = 81)	Recognition threshold for sweet (sucrose), sour (tartaric acid), salt (sodium chloride), bitter (quinine hydrochloride)	No significant between-group differences for sweet, sour, salt, bitter. Patients who received chemotherapy alone had a significantly higher salt taste threshold compared with chemotherapy + radiation and/or HSCT (no effect on sweet, sour, or bitter). No significant impact of duration since treatment (<5 y vs ≥5 y)
Skolin et al, 2006^8^ Cross-sectional design	Sweden	Children receiving treatment^c^ (n = 10); median age: 14.5 y Leukemia (n = 9); solid tumor (n = 6); lymphoma (n = 5); CNS tumor (n = 2)	Chemotherapy: doxorubicin, methotrexate, ifosfamide, cytarabine, procarbazine/dacarbazine, cisplatin and cyclophosphamide; distribution of drugs not specified	Healthy non-nicotine using volunteers (n = 10); age of control participants not specified	Recognition threshold for sweet (sucrose), sour (citric acid), salt (sodium chloride), bitter (quinine hydrochloride)	Patients had significantly higher recognition thresholds for bitter compared with control participants. No significant between-group differences were observed for sweet, sour, or salt tastes.
Tanem et al, 2022^35^ Cross-sectional design	Norway	Childhood cancer survivors^d^ (n = 40); mean age): 28.9 y MB (n = 35); CNS-PNET (n = 5)	Chemotherapy and radiotherapy; type not specified	Identification of ≥ 2 concentrations (sweet, sour, salty) and ≥ 1 concentration (bitter) was considered normal. Identification of ≥ 9 concentrations (all tastants), normal; <9, hypogeusia; no sensation, complete taste impairment; based on normative adult data (n = not specified)	Recognition threshold for sweet (sucrose), sour (citric acid), salt (sodium chloride), bitter (quinine hydrochloride)	Thirteen (32.5%) participants had hypogeusia. Nine participants (22.5%) were ageusic for ≥1 taste qualities (sour, n = 5; and salt, n = 4; most common). Survivors scored significantly lower on identification of sweet, sour, and salt tastes compared with normative data.
Trogdon Wall and Gabriel 1983^36^ Cross-sectional design	United States	Children with cancer in remission or relapse (n = 26); age: most were ≤ 7 y old (n = 17) ALL (n = 26)	Chemotherapy; type not specified	Children free of disease, not taking medications (n = 23)	Detection and recognition threshold for sweet, sour, salt, bitter	Patients had significantly higher detection thresholds for sweet and salt taste, and significantly higher recognition threshold for sweet, salt, sour, and bitter, compared with control participants.
Van den Brink et al, 2021^37^ Prospective design	The Netherlands	Children receiving treatment (n = 31); median age: 12 y Hematology malignancy; ALL (n = 8); AML (n = 1); lymphoma (n = 7); brain/solid tumor; medulloblastoma (n = 3); bone (n = 9); rhabdomyosarcoma (n = 3)	Chemotherapy: alkylating (n = 14); anthracycline (n = 7); platinum (n = 4); *Vinca* alkaloids (n = 15); antimetabolites (n = 11); epipodophyllotoxins (n = 5); other (n = 11)	Healthy siblings and friends of patients (n = 24); and change over a single cycle of chemotherapy (median time between assessments: 21 d)	Recognition threshold for sweet (sucrose), sour (citric acid), salt (sodium chloride), bitter (quinine hydrochloride)	Patients had significantly different sour taste scores compared with control participants. No significant between-group differences for sweet, salt or bitter tastes. Sweet, bitter, and total taste scores were significantly higher after a cycle of chemotherapy.

a Minimum 5 years since treatment completion.

b Minimum 6 months since treatment completion.

c The number of participants for taste acuity testing.

d Minimum of 2 years since treatment completion.

*Abbreviations:* ALL, acute lymphoblastic leukemia; AML, acute myeloid leukemia; AMML, acute monomyelogenous leukemia; CNS-PNET, central nervous system supratentorial primitive neuroectodermal tumor; HSCT, hematopoietic stem cell transplant; MB, medulloblastoma.

In the hedonics search arm, data from 160 children with cancer were examined (Table 4^23^^,^^25^^,^^31^^,^^36^). Hematological cancers were the most prevalent cancer type. However, in an article by Bernstein,^25^ cancer type was not defined. All children received chemotherapy as the primary treatment type. The hedonic factors under investigation varied between the studies, including appetite^23^^,^^36^ and food liking or aversions.^25^^,^^31^^,^^36^ Study control participants were either healthy children, children not receiving chemotherapy known to cause gastrointestinal (GI) toxicity, or a comparison to pretreatment conditions.

**Table 4:** Characteristics and findings from studies that investigated hedonic experiences in children with cancer and in cancer survivors

Reference; study design	Country	Participants and cancer type	Treatment	Control	Hedonic factor assessed and method	Key findings
Ameringer et al, 2013^23^ Longitudinal, descriptive pilot design	United States	Children receiving treatment (n = 9); mean age: 15.3 y Bone tumor (n = 3); leukemia (n = 2); lymphoma (n = 3); soft tissue tumor (n = 1); no further treatment details specified	Chemotherapy, type not specified	Change over a single cycle of chemotherapy (own control): day 1 of a chemotherapy cycle, day 2 of chemotherapy, 7–10 d after the after the start of a chemotherapy cycle, and day 1 of the next chemotherapy cycle	Appetite assessed with a 100-mm visual analog scale, with anchors of 0 (normal) to 100 (no appetite at all). Rating of worst level of appetite in last 24 h	67% of patients reported decreased appetite on day 1 of a chemotherapy cycle, and 33% of patients reported decreased appetite at day 1 of the next chemotherapy cycle. No significant differences were found in mean appetite severity within a single cycle of chemotherapy.
Bernstein et al, (1978)^25^ Prospective cohort design	Not stated	Children receiving treatment (n = 41); age: 2–6 y Cancer type not specified	Chemotherapy, type not specified	Experimental group: unusual ice cream flavor and chemotherapy Control groups: chemotherapy + no ice cream or vincristine (non-GI toxic chemotherapy)/no chemotherapy + unusual ice cream flavor	Learned food aversion Likelihood of selecting the novel food 2–4 wk after initial exposure	Consumption of a novel food prior to chemotherapy significantly decreased likelihood of consumption of same food when offered again (21%), compared with those who had not previously been offered the item (67%) or those with non-GI toxic chemotherapy + novel “scapegoat” food (73%).
Bernstein et al, 1982^31^ Prospective cohort design	United States	Children receiving treatment (n = 84); aged 2–18 y ALL, lymphoma, AML, Wilms’ tumor, Ewing’s sarcoma, Hodgkin’s disease Distribution of cancer types not specified	Chemotherapy, type not specified	Experimental group: chemotherapy ± unusual ice cream exposure prior to treatment Control group: vincristine (non-GI toxic chemotherapy) or no treatment	Learned food aversion Comparison of pretherapy dietary questionnaire and second questionnaire at least 1 wk later. Aversions present when foods consumed prior to therapy were no longer preferred, actively disliked, or no longer listed as consumed in usual intake.	Patients receiving chemotherapy associated with GI toxicity reported aversions more often than did control participants. GI-toxic chemotherapy alone was associated with incidence of aversions. When GI-toxic chemotherapy was paired with an unusual ice cream flavor, there were no between-group differences.
Trogdon Wall and Gabriel, 1983^36^ Cross-sectional design	United States	Children with cancer in remission or relapse (n = 26); age: most were ≤ 7 y old (n = 17) ALL (n = 26)	Chemotherapy; type not specified	Children free of disease, not taking medications (n = 23)	Food liking and appetite Parent-completed, 14-item questionnaire and 62-item food checklist to assess likes and dislikes before illness and current (or currently liked or disliked, for control participants) 5-point scale to assess appetite	Patients liked food in the meat group significantly less than did control participants. Majority of changes in food liking were in the salt category (direction of change not specified) No significant differences in appetite rating were found between patients and control participants.

*Abbreviations:* ALL, acute lymphoblastic leukemia; AML, acute myeloid leukemia; ANLL, acute monomyelogenous leukemia; GI, gastrointestinal; NS, not stated.

### Alterations in taste perception related to cancer treatment

#### Sour taste perception

In 50% of studies (n = 2 of 4), significantly higher sour-taste recognition thresholds were found in children receiving chemotherapy (*t*_37_ = –2.74; *P* < 0.05) or undergoing BMT (6.09 mmol/L vs 4.5 mmol/L; *P* = 0.006) compared with healthy control children. One study found that children receiving chemotherapy had a different sour taste score compared with healthy control participants (threshold values not provided; *P* = 0.042).^30^^,^^36^^,^^37^ Alternatively, 1 study found no significant difference in recognition thresholds between children receiving chemotherapy and healthy control participants.^8^

Considering changes in taste perception across treatment, 3 studies observed no significant changes in sour taste thresholds between pre- and post-treatment cycles (chemotherapy or BMT).^30^^,^^32^^,^^37^ However, Majorana et al^33^ determined that sour taste sensitivity had decreased from pretreatment to during conditioning therapy for BMT (0.000032 M [lowest concentration] at baseline vs 0.00032 M during conditioning therapy) and remained decreased after engraftment (0.0001 M; *P* = 0.02).

In survivors of childhood cancer (mean time since treatment, 12.4 years; range, 5–38 years), prevalence of sour taste dysfunction was 15.7%.^14^ Tanem et al^35^ found that survivors (mean time since treatment, 20.5 years; range, 3.5–40.4 years) were able to correctly identify fewer concentrations of a sour tastant, compared with healthy adults (normative data; 2.0 vs 3.0; *P* < 0.001). However, no significant differences were determined between survivors (median time since treatment, 6.5 years; range, 6 months to 3 years) and healthy control participants in another study.^34^ Findings for sour taste perception were varied. Children receiving cancer treatment are likely to experience altered sour-taste perception; however, it remains unclear whether such alterations remain after treatment and into survivorship.

#### Bitter taste perception

In 50% of studies (n = 2 of 4), children with cancer had a higher bitter taste recognition threshold during chemotherapy than did control participants (0.015 mm/L vs 0.004 mm/L quinine hydrochloride, *P* < 0.05; and *t* = –2.26, *P* < 0.05, respectively).^8^^,^^36^ Notably, the most common taste recognition error made by children with cancer was the identification of bitter taste when another tastant was present.^8^ Compared with control participants, children incorrectly reported a bitter tastant most frequently (*P* < 0.05).^8^ However, no significant differences in bitter taste perception were observed between children with cancer and healthy control participants in 2 other studies (*P* > 0.05).^30^^,^^37^

No significant changes were observed for bitter taste thresholds between pre- and post-treatment cycles in 2 studies.^30^^,^^32^ Majorana et al^33^ demonstrated that bitter-taste sensitivity was lower during conditioning therapy for BMT compared with pretreatment (0.000032 M [lowest concentration] at baseline vs 0.00032 M during conditioning therapy) but returned to pretreatment thresholds after engraftment (*P* = 0.03). Van den Brink et al^37^ demonstrated that bitter-taste scores were higher after 1 cycle of chemotherapy compared with day 1 of the cycle (threshold values not provided; *P* = 0.028), meaning that the bitter tastant was correctly identified more often after chemotherapy.

In survivors of childhood cancer (mean time since treatment, 12.4 years; range, 5–38 years), prevalence of bitter taste dysfunction was 11.8%.^14^ No significant differences were observed between survivors of childhood cancer and healthy control participants in 2 studies.^34^^,^^35^ Childhood cancer treatment inconsistently influenced bitter-taste perception in children during cancer treatment, but any alterations to perception may not remain into survival.

#### Sweet taste perception

During chemotherapy, in 75% of studies (n = 3 of 4), researchers observed no significant differences in sweet-recognition thresholds between children with cancer and control participants.^8^^,^^30^^,^^37^ Alternatively, 1 study demonstrated that sweet-taste detection and recognition thresholds were significantly higher (*t* = –2.36 and *t* = –2.56, respectively; *P* < 0.05) compared with those of control participants.^36^

Two studies found no significant differences in sweet-taste thresholds between admission and after BMT.^30^^,^^32^ However, van den Brink et al^37^ observed that sweet-taste scores were significantly higher after the commencement of chemotherapy (median interval between tests, 21 days; *P* < 0.001; threshold values not provided). Majorana et al^33^ demonstrated that children could detect sweet taste at the lowest concentration (0.000032 M) pretreatment. During conditioning therapy, sweet-taste sensitivity decreased (0.0001 M), but taste function returned to baseline after engraftment (*P* = 0.02).^33^

Among cancer survivors, prevalence of impaired sweet-taste perception was 9.8% (mean time since treatment, 12.4 years; range, 5–38 years).^14^ Tanem et al^35^ observed that taste function did not recover after cancer treatment. Survivors of childhood cancer (mean time since treatment, 20.5 years; range, 3.5–40.4 years) could correctly identify fewer concentrations of a sweet tastant compared with healthy adults (normative data; 2.9 vs 3.3; *P* = 0.035).^35^ Alternatively, Nagai et al^34^ found no significant differences in sweet-taste thresholds between survivors (median time since treatment, 6.5 years; range, 6 months to 13 years) and a healthy control group. During cancer treatment, taste perception was unlikely to be altered; however, findings for cancer survivors are inconclusive.

#### Salt taste perception

During chemotherapy, 75% of studies (n = 3 of 4) demonstrated no significant differences in salt taste perception between children with cancer and healthy control participants.^8^^,^^30^^,^^37^ Conversely, 1 study demonstrated salt taste detection and recognition thresholds were higher in children with cancer during chemotherapy, compared with control participants (*t* = –3.01 and *t* = –2.99, respectively; *P* < 0.05).^36^

When assessing salt taste perception across treatment, 2 studies observed no significant changes in salt taste thresholds between pre- and post-treatment cycles (chemotherapy or BMT).^32^^,^^37^ Barale et al^30^ demonstrated that salt taste thresholds were significantly higher 2 days after transplant compared with at admission (mean paired difference: –40.5; *P* = 0.016); however, thresholds returned to admission values at 45 days after transplant.^30^ During conditioning therapy for BMT, Majorana et al^33^ demonstrated salt taste sensitivity decreased compared with pretreatment (0.000032 M [lowest concentration] at baseline vs 0.00032 M during conditioning therapy) and remained decreased after engraftment (0.0001 M; *P* < 0.01).^33^

In survivors of childhood cancer (mean time since treatment, 12.4 years; range, 5–38 years), salt taste dysfunction was only present in 7.8% of survivors.^14^ Tanem et al^35^ demonstrated that survivors (mean time since treatment, 20.5 years; range, 3.5–40.4 years) could correctly identify fewer concentrations of a salt tastant, compared with healthy adults (normative data; 2.4 vs 3.1; *P* = 0.003). Nagai et al^34^ found no significant differences in salt taste threshold differences between survivors (median time since treatment, 6.5 years; 6 months to 13 years) and healthy control participants.^34^ However, salt taste threshold was significantly higher in survivors who had received only chemotherapy alone compared with multimodal treatments (chemotherapy plus radiation and/or hematopoietic stem cell transplant; values not specified; *P* = 0.02).^34^ Salt taste perception was minimally affected by childhood cancer treatment; however, findings for survivors were mixed.

### Food liking and aversion

Exposure to chemotherapy agents known to cause GI toxicity was associated with the development of learned food aversions. Children exposed to a novel food (namely, an unusual ice cream flavor) 15–60 minutes prior to GI-toxic chemotherapy demonstrated significantly lower preference for that food when it was offered again 2–4 weeks later, compared with a group of children who were either not receiving GI-toxic chemotherapy or who were not exposed to the novel food originally (*z* = 3.06; *P* ≤ 0.001).^25^ In the case of familiar foods (consumed 4–5 hours prior to chemotherapy), food aversions occurred more often in children receiving GI-toxic chemotherapy (62.5%) than in control participants (35.4%) who received non–GI-toxic chemotherapy, when assessed 1 week after exposure (*P* < 0.02).^31^

Only 1 study investigated changes in food liking.^36^ Compared with healthy control participants, children with cancer liked meat (eg, beef, hamburger, lunch meats beans, poultry, fish) less (*t*_47_ = –2.69; *P* < 0.0125).^36^ When foods were grouped according to their dominant taste quality, the majority of changes to food liking were in the salt category (direction of change and data not shown). However, it should be noted that many meat foods (eg, sausages, hot dogs, and lunch meats) were included in the salt category, which may influence a negative change ^36^

### Appetite

Two studies investigated the influence of chemotherapy on appetite. Children reported decreased appetite prior to the start of a chemotherapy cycle (67% on day 1 of a cycle; 33% on day 1 of the following cycle).^23^ However, there was no significant difference in mean severity of appetite loss at the 2 different times within a single chemotherapy cycle.^23^ Trogdon Wall and Gabriel^36^ found no significant differences in appetite between children with cancer and control study participants. However, parents reported that children with cancer had to be “coaxed to eat” more frequently than did healthy children (χ^2^ = 7.58; *P* < 0.05).

## DISCUSSION

Treatment-related side effects such as changes in taste perception and hedonics may affect dietary intake by children. The findings of this systematic review provide evidence that childhood cancer treatment does not influence taste perception in a uniform direction. Sour, bitter, sweet, and salt tastes were all influenced by childhood cancer treatment in a varied and unpredictable fashion. Notably, umami (the “fifth taste”) was not examined in the literature. Alterations to taste perception were observed after cancer treatment; however, findings were inconclusive and derived from cross-sectional data. Regarding hedonic factors, chemotherapy agents known to cause GI toxicity were associated with learned food aversions in children, a side effect that may remain months after initial exposure. The effects of cancer treatment on appetite were also inconclusive. The notable variable effects of cancer treatments on taste perception and hedonics highlight the importance of individualized monitoring, assessment, and treatment of these side effects in clinical practice.

Findings regarding taste perception during childhood cancer treatment were mixed, consistent with previously published reviews.^17^^,^^26^ In adults with cancer, changes in taste perception have been reported as overall varied and cyclical with some unpredictability.^17^^,^^26^ As such, these impacts are unlikely to be completely due to a direct effect of cancer treatment on taste perception alone but may involve other hedonic (eg, appetite), and sensory components of flavor (eg, smell, touch, and chemesthesis). Alternatively, phenomena such as phantom metallic taste may have a greater influence on how foods are perceived during and after childhood cancer treatment.^38^ Researchers should continue to examine these sensory and hedonic influence of childhood food choices during cancer and after cancer treatment.^38^

Subjective measures, including self- or parent reports, are commonly used to assess taste perceived by children with cancer and may better capture the influence of hedonics, flavor, or phantom tastes on food and eating behaviors. According to subjective self-reporting of patients and their parents, altered taste is the predominant cause of decreased or altered food intake in children undergoing cancer treatment.^8^ As such, these subjectively assessable factors are likely to have clinical relevance and impact on food intake and nutritional status of children with cancer.

In this review, we provide evidence that sour and bitter tastes were most affected during childhood cancer treatment. Additionally, children with cancer incorrectly reported a bitter taste when presented with another tastant (ie, sweet or salt) or water. The phantom presence of bitter and sour oral sensations were reported previously in a study,^39^ with 25% and 12% of children undergoing cancer treatment report the food tastes bitter or sour, respectively. Such symptoms were reported to result in decreased food intake and enjoyment.^39^ Being less sensitive to sour and bitter tastes may be hypothesized as beneficial to the dietary intake of children. For instance, lower sensitivity to the bitter compounds, such as 6-*n*-propylthiouracil, have been shown to enhance consumption of bitter foods such as cruciferous vegetables and dietary fat.^40^ Although more studies are required to confirm the effects of treatment on taste sensitivity, it is equally important to investigate other taste-related phenomena and the impact they have on children’s nutritional status.

To our knowledge, no studies have investigated umami taste perception by children or survivors of childhood cancer. Umami taste is considered difficult to assess in children because of a lack of children’s familiarity with the concept and the challenges in describing it.^32^ Umami taste is of particular relevance to children with cancer, because of the high protein content of umami-rich foods (eg, meat, poultry, and soy products) and the importance of protein for growth and development. We also found that liking of meat (eg, beef, beans, poultry, and fish) was reduced in children who were undergoing chemotherapy.^36^ Therefore, altered umami taste perception may clarify these changes in food enjoyment and intake. Researchers should consider age-appropriate measures of umami taste perception and determine its influence on intake of protein-rich foods in children with cancer.

We also found in this review that altered taste perception affected a moderate percentage of survivors of childhood cancer, up to 40 years after treatment. Childhood cancer survivors are at increased risk of chronic conditions, including obesity and the metabolic syndrome.^41^ In part, these chronic conditions are hypothesized to be due to poor diet quality and poor adherence to healthy eating guidelines.^42^^,^^43^ In previous studies, 32%–50% of childhood cancer survivors consumed less than the recommendations for folate, calcium, and iron,^42^ as well as nutrient-rich foods such as vegetables and fruits.^43^ However, the assessment of taste function in cancer survivors was primarily examined using cross-sectional methods, and thus it is important for longitudinal research to examine the trajectory of taste function across treatment and survivorship. Additionally, the impact of childhood cancer treatment on dietary intake in survivors is likely to be complex. Although we found no studies in which hedonic factors in childhood cancer survivors were assessed, food aversions and selective eating are potential barriers to achieving a healthy dietary intake. Notably, parent-reported picky eating behaviors are more common among survivors and picky eating, emotional overeating, and body mass index have been shown to greatly effect diet quality.^44^ More longitudinal research is required to determine whether and to what extent taste and/or hedonic factors effect childhood cancer survivors and influence food consumption.

### Strengths and limitations

To our knowledge, this review is the first of its type to summarize the effects of cancer treatment on taste perception and food hedonics in children with cancer or childhood cancer survivors. A rigorous search strategy and literature summary were developed, following the PRISMA statement.^28^ This 2-armed systematic review reflected and built upon that conducted by Boltong et al,^17^ extending current knowledge pertaining to adults to the pediatric population. Overall, the literature was limited, with few eligible studies of small sample sizes and heterogeneity in regard to cancer types, treatment regimens, and outcome assessment methods. This heterogeneity limits our ability to make robust conclusions and targeted recommendations for specific cohorts of patients with childhood cancer. The quality of eligible studies was variable, with half rated neutral to negative. Given the small and well-known body of literature in this field, articles on studies that examined the influence of cancer treatment other than radiotherapy and chemotherapy were identified in a snowball search and were subsequently included because of the apparent effects of other treatments on taste perception and food hedonics.

### Potential implications and future directions

This review revealed a paucity of studies investigating the effects of childhood cancer treatment on factors that influence food intake, namely, taste perception and food hedonics. Factors other than taste and food hedonics that contribute to the overall eating experience, including other sensory inputs such as smell dysfunction, flavor, and oral sensations such as metallic taste or phantom bitter taste, should be considered in further research. We found that umami taste was not investigated in the literature and may also be affected by childhood cancer treatment. Because of the umami-rich nature of protein-containing foods, studies assessing umami taste perception in patients with childhood cancer and survivors of childhood cancer could be important for the prevention of malnutrition in children with cancer. In all, more high-quality, longitudinal studies in which data are collected at multiple time points (eg, before, during, and after treatment) are required to determine the magnitude of taste and hedonic changes in treatment and survivorship. Moreover, greater understanding of these changes is required to determine the psychophysical factors of food choice and eating behaviors. This information will support the development of effective assessment frameworks and practical nutritional interventions to support good nutritional intake and status into survivorship.

### Conclusion

In this 2-armed systematic review, we summarized current evidence on 2 nutrition-related phenomena in children with cancer and survivors of childhood cancer: taste perception and food hedonics. Although there is some evidence to suggest alterations to taste perception occur in children with and survivors of childhood cancer, these findings are inconsistent and so limit the ability to design interventions including specific clinical recommendations. Learned food aversions and changes to food liking occurred during cancer treatment, but impact on appetite was understudied and findings were varied. Currently, the varied effects of cancer treatments on taste perception and hedonics support an individualized approach to monitoring and assessment of these side effects and personalized nutritional interventions. Large, multicenter studies in a range of childhood cancer treatments may enable specific conclusions to be drawn on taste perception and food hedonics. These data are key to underpinning the formation of assessment frameworks and practical nutritional interventions for children during and beyond cancer treatment.

## Supplementary Material

nuad162_Supplementary_Data

## Supporting Information

The following Supporting Information is available through the online version of this article at the publisher’s website.

*Table S1* **PRISMA checklist**

*Table S2* **Medline complete search strategy (taste search arm)**
