# Supplementary material for: The influence of pediatric cancer treatment on taste perception and food hedonics: a systematic review
Source: Nutr Rev. 2024 Jan 10;82(12):1741–55. doi: 10.1093/nutrit/nuad162 (PMC11551449; doi:10.1093/nutrit/nuad162)
Supplement: nuad162_Supplementary_Data [file nuad162_supplementary_data.zip › nuad162_Supplementary_Data/Curtis_Supplementary Table2_3.docx]

| **Supplementary Table 2.** Medline Complete search strategy (taste search arm) | |
| --- | --- |
| 1 | TI chemotherapy OR AB chemotherapy |
| 2 | TI radiotherapy OR AB radiotherapy |
| 3 | TI "radiation therapy" OR AB "radiation  therapy" |
| 4 | (MH "Drug Therapy") OR (MH "Consolidation  Chemotherapy") OR (MH  "Chemoradiotherapy+") OR (MH  "Chemotherapy, Adjuvant") |
| 5 | MH "Radiotherapy+") OR (MH  "Radiotherapy, Adjuvant") |
| 6 | TI taste OR AB taste |
| 7 | (MH "Taste") |
| 8 | S6 OR S7 |
| 9 | S1 OR S2 OR S3 OR S4 OR S5 |
| 10 | S8 AND S9 |
| Abbreviations: TI, title; AB, abstract; MH, MESH heading | |

| **Supplementary Table 3.** Medline Complete search strategy (hedonics search arm) | |
| --- | --- |
| 1 | TI chemotherapy OR AB chemotherapy |
| 2 | TI radiotherapy OR AB radiotherapy |
| 3 | TI "radiation therapy" OR AB "radiation  therapy" |
| 4 | (MH "Drug Therapy") OR (MH "Consolidation  Chemotherapy") OR (MH  "Chemoradiotherapy+") OR (MH  "Chemotherapy, Adjuvant") |
| 5 | MH "Radiotherapy+") OR (MH  "Radiotherapy, Adjuvant") |
| 6 | TI liking OR AB liking |
| 7 | TI appetite OR AB appetite |
| 8 | (MH "Appetite") |
| 9 | TI hedonic* OR AB hedonic* |
| 10 | S6 OR S7 OR S8 OR S9 |
| 11 | S1 OR S2 OR S3 OR S4 OR S5 |
| 12 | S10 AND S11 |
| Abbreviations: TI, title; AB, abstract; MH, MESH heading | |
